# Dynamic risk stratification in patients with follicular thyroid carcinoma treated with lobectomy

**DOI:** 10.1530/ETJ-25-0154

**Published:** 2025-11-17

**Authors:** Haruhiko Yamazaki, Kiminori Sugino, Ryohei Katoh, Kenichi Matsuzu, Wataru Kitagawa, Mitsuji Nagahama, Aya Saito, Koichi Ito

**Affiliations:** ^1^ Department of Breast and Thyroid Surgery, Yokohama City University Medical Center, Yokohama City, Kanagawa, Japan; ^2^ Department of Surgery, Ito Hospital, Tokyo, Japan; ^3^ Department of Pathology, Ito Hospital, Tokyo, Japan; ^4^ Department of Surgery, Yokohama City University School of Medicine, Yokohama City, Kanagawa, Japan

**Keywords:** dynamic risk stratification, follicular thyroid carcinoma, prognosis

## Abstract

**Background:**

Previous studies showed that dynamic risk stratification (DRS) was also useful in differentiated thyroid carcinoma patients with lobectomy or total thyroidectomy without radioactive iodine. The aim of this study was to evaluate the DRS system in patients with follicular thyroid carcinoma (FTC) who underwent lobectomy alone.

**Methods:**

In total, 161 patients with FTC who were diagnosed between January 2005 and December 2014 and underwent lobectomy alone were included in this study.

**Results:**

Of the 161 patients with FTC, the DRS system classified 159 patients (99%) as having an excellent response, and 2 (1%) as having a structural-incomplete response. The 10-year disease-free survival (DFS) rates of patients with excellent response and structural-incomplete response were 93.5% and 0%, respectively (*P* < 0.001). Of the 54 patients with VI ≥ 2, the 10-year DFS rates of patients with excellent response (*n* = 53) and structural-incomplete response (*n* = 1) were 83.2% and 0%, respectively (*P* < 0.001). Of the 53 patients with excellent response, 10 patients experienced disease recurrence. Among Of these ten patients, the duration between initial thyroid surgery and recurrence was 2–5 years in one, 5–8 years in two, 8–10 years in four, and 10 years or more in three patients, respectively.

**Conclusion:**

Long-term follow-up may be needed in FTC patients treated with lobectomy alone even though they had an excellent response to initial thyroid surgery, especially in patients with a higher risk of recurrence.

## Introduction

Thyroid carcinoma is the most common endocrine malignant tumor, and differentiated thyroid carcinoma (DTC), which is composed of papillary thyroid carcinoma (PTC) and follicular thyroid carcinoma (FTC), accounts for approximately 95% of all thyroid carcinomas (1, 2). Since almost all DTCs have a good prognosis, recent clinical practice guidelines recommend a risk-adapted treatment strategy for individual patients (3, 4). The American Thyroid Association (ATA) initial risk stratification system appropriately predicts the risk of recurrence or persistent disease (3). However, many DTC patients are initially categorized as ATA intermediate-risk, which has varied clinical outcomes. Therefore, the ATA initial risk stratification, which is composed of clinicopathological factors and genetic information, has some limitations in its applicability to appropriate individual patient management, and further appropriate risk stratification tools are desired.

Dynamic risk stratification is defined as the best response to total thyroidectomy (TT) and radioactive iodine (RAI) therapy during the first 2 years of follow-up (5). The DRS system classifies patients into the following four response categories based on clinical, biochemical, and imaging findings: excellent, indeterminate, biochemical-incomplete, and structural-incomplete. The DRS system probably allows physicians to provide more appropriate follow-up for individual patients (6). Although the DRS system has been validated for patients with DTC treated with TT and RAI, previous studies have proposed that DRS may also be used in patients with lobectomy or TT without RAI (7, 8).

FTC is the second most common histological type of thyroid carcinoma and is divided into the following three categories by the fifth edition of the World Health Organization (WHO): minimally invasive follicular thyroid carcinoma (MI-FTC), encapsulated angioinvasive follicular thyroid carcinoma (EA-FTC), and widely invasive follicular thyroid carcinoma (WI-FTC) (9). In general, most patients with FTC first undergo hemithyroidectomy with a diagnosis of follicular or adenomatous nodules, and completion TT following RAI is considered in FTC with risk factors for recurrence (10). Since the incidence of FTC is low at approximately 5% in comparison to PTC, the majority of histological types were PTC in previous studies that indicated the utility of ATA risk stratification and DRS (5). In fact, the incidence in previous studies that indicated the utility of DRS for DTC patients with lobectomy or TT without RAI, including FTC, only ranged from 0 to 5.5% (7, 8, 11, 12). In terms of predicting the prognosis of FTC, the histological subtype or degree of vascular invasion is important (13). However, these factors have not been considered in previous studies. Therefore, it is uncertain whether DRS is truly useful for predicting disease recurrence or follow-up among patients with FTC who underwent lobectomy or TT without RAI.

The aim of this study was to evaluate the DRS system in patients with FTC who underwent lobectomy alone, incorporating pathological classification based on the latest WHO classification.

## Materials and methods

### Ethics approval

The study protocol was approved by the Ethics Review Board (approval no. 466). The study was performed in accordance with the 1964 Declaration of Helsinki and its later amendments. The ethics review board waived the requirement for informed consent due to the nature of the retrospective cohort study.

### Study participants

**Figure 1:**
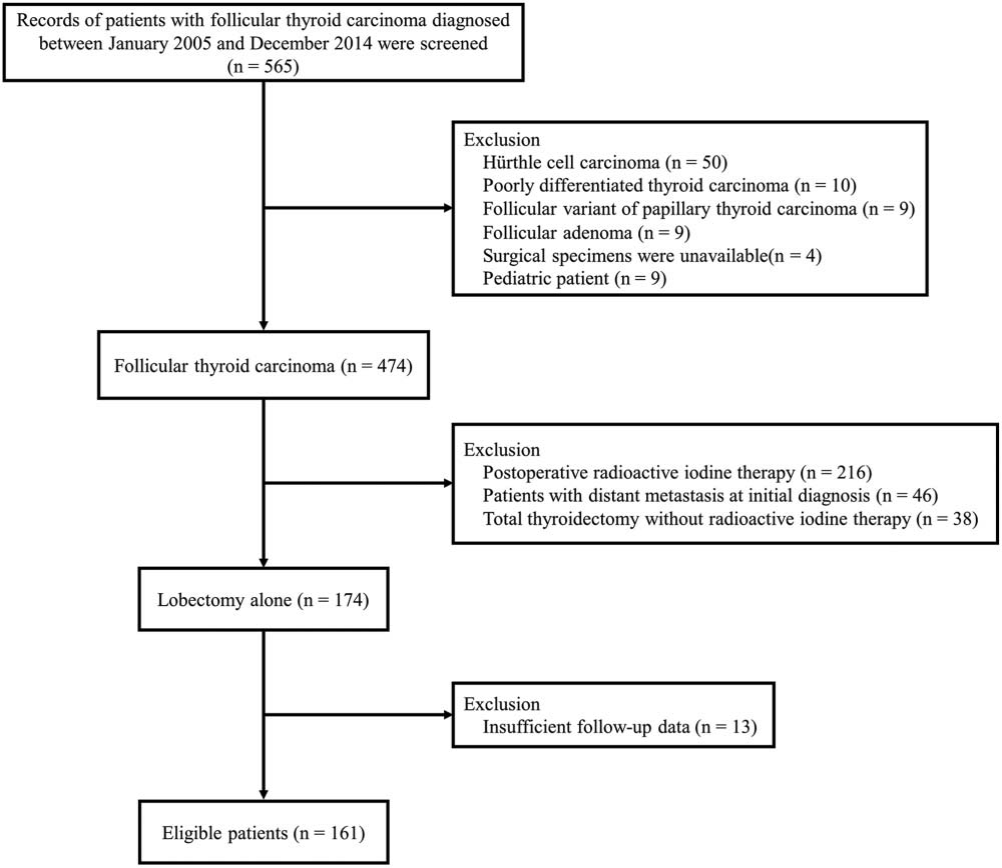
Enrollment and participation flow diagrams.

Figure 1 shows enrollment and participation flow diagrams. A total of 565 patients were diagnosed with FTC between January 2005 and December 2014. During this period, FTC was diagnosed based on the third edition of the WHO classification (14). After reviewing the pathological specimens based on the fifth edition of the WHO classification by one pathologist with a special interest in thyroid neoplasia (R K), 474 patients with FTC were included in our previous study (9, 13). Of these 474 patients, we reviewed the medical records of 174 with FTC who underwent lobectomy alone as initial thyroid surgery. Of these 174 patients, 13 did not have sufficient follow-up duration or laboratory data. After excluding these 13 patients, 161 patients with FTC were included in this study. The median follow-up period was 11.0 years. In our hospital, RAI therapy was recommended for patients aged ≥45 years or with WI-FTC during this study period (15).

### Pathological analyses

We previously explained the details of the pathological analysis and evaluation of VI (13, 16). EA-FTC was defined as a solid nodule in the thyroid gland that was completely encapsulated, and an intravascular tumor plug found within the fibrous capsule was clearly demonstrated to be covered by the endothelium (13). WI-FTC was defined as carcinoma that shows extensive invasion of the thyroid and extra-thyroid soft tissue (17). The sections were stained with hematoxylin and eosin. Immunohistochemical staining for CD31 and CD34 was used to distinguish VI from pseudoinvasion when needed. Furthermore, immunohistochemical staining for D2-40 was used to distinguish VI from lymphatic invasion in select cases.

### Follow-up

After the initial thyroid surgery, patients were usually followed every 3 months during the first year and at 6–12-month intervals thereafter at the discretion of the attending physician based on the risk of the individual patient and the clinical course of the disease. Neck ultrasonography (US) was performed annually. Systemic examinations, such as computed tomography (CT), bone scintigraphy, and whole-body scan (WBS), were performed only when clinicians considered recurrence.

### Stratification

Initial stratification was performed using the WHO classification (9), ATA risk stratification (3), and degree of VI (13). We categorized MI-FTC with ≤ T2 as ATA low-risk, EA-FTC with VI > 4 and WI-FTC as ATA high-risk, and others as ATA intermediate-risk since no one had an intermediate-high-risk category. Receiver operating characteristic analysis showed an optimal cutoff VI of 2 for cause-specific survival (CSS) (area under the curve (AUC) = 0.77 (sensitivity: 0.80; specificity: 0.59)) and disease-free survival (DFS) (AUC = 0.76 (sensitivity: 0.85; specificity: 0.66)) in our previous study (13). Therefore, a cutoff VI of 2 was also used in this study. Patients were re-stratified according to their response to therapy assessment using the ATA 2025 guideline definitions as having an excellent or structural-incomplete response to therapy (3). The best response during the first 2 years of follow-up was used to define the response to initial therapy (Table 1).

**Table 1:** Response to therapy definitions post-lobectomy.

Excellent	Normal or low-risk nodules in the contralateral lobe, or contralateral lobe nodules with benign biopsy and no abnormal lymph nodes on imaging
Indeterminate	Not applicable
Biochemical incomplete	Not applicable
Structure incomplete	Structural evidence of disease (suspicious imaging or biopsy proven local or distant metastatic disease)

### Definitions

The final clinical outcomes were defined as follows: no structural evidence of disease (SED), recurrent/persistent SED, or death from FTC (7). CSS was calculated as the duration from the point of diagnosis to death from FTC. DFS was calculated as the duration from diagnosis to any structural recurrence.

### Statistical analysis

All statistical analyses were conducted using EZR (Saitama Medical Center, Jichi Medical University, Saitama, Japan), which is a graphical user interface for R (The R Foundation for Statistical Computing, Vienna, Austria) (18). The Kaplan–Meier method was used to construct CSS and DMFS curves. Statistical significance was set at *P* < 0.05. The final examination date for the survival analysis was confirmed using medical records.

## Results

### Baseline patient characteristics

The baseline characteristics of the patients are summarized in Table 2. Of the 161 patients with FTC, 35 (22%) were male and 126 (78%) were female. The median age at diagnosis was 37 years (interquartile range (IQR): 29–56 years), the median tumor size was 44 mm (IQR: 30–55 mm), and the median thyroglobulin (Tg) concentration measured before the initial thyroid surgery was 608 ng/mL (IQR: 108–1,565 ng/mL), and 54 (34%) patients had VI ≥ 2. According to the WHO classification, 55 (34%) patients had MI-FTC, 101 (63%) had EA-FTC, and 5 (3%) had WI-FTC. According to ATA risk stratification, 28 (17%) had low-risk, 103 (64%) had intermediate-risk, and 30 (19%) had high-risk disease. Of the 161 patients, 54 patients did not undergo completion thyroidectomy and RAI therapy even though they were aged ≥45 years (*n* = 49) or had WI-FTC histology (*n* = 5). The reasons were as follows: patient’s preference (*n* = 21), decrease of Tg level (*n* = 15), comorbidity including cerebrocardiovascular disease or advanced age (*n* = 8), physician’s choice (*n* = 3), tumor size <10 mm (*n* = 2), benign nodule as initial diagnosis (*n* = 1), and unknown (*n* = 4). In these 54 patients, distant structural disease had been excluded by pre- or post- operative CT.

**Table 2:** Baseline characteristics. Data are presented as *n* (%) or median (IQR).

Characteristics	Values
Total *n*	161
Sex	
Male	35 (22%)
Female	126 (78%)
Age (years)	37 (29–56)
Age (years)	
<55	119 (74%)
≥55	42 (26%)
Histological subtype	
Minimally invasive	55 (34%)
Encapsulated angio-invasive	101 (63%)
Widely invasive	5 (3%)
ATA risk stratification	
Low	28 (17%)
Intermediate	103 (64%)
High	30 (19%)
Number of vascular invasions	1 (0–2)
Vascular invasion	
Yes	104 (65%)
No	57 (35%)
Number of vascular invasions	
<2	107 (66%)
≥2	54 (34%)
Tumor size (mm)	44 (30–55)
Tumor size (mm)	
≤40	73 (45%)
>40	88 (55%)
Tg (ng/mL)	608 (108–1,565)
TgAb	
Yes	17 (11%)
No	144 (89%)

ATA, American Thyroid Association; IQR, interquartile range; Tg, thyroglobulin; TgAb, thyroglobulin antibody.

### Clinical outcomes

Of the 161 patients with FTC, 4 patients experienced local recurrence, 10 patients experienced both local and distant metastatic recurrence, and 1 patient experienced death from FTC after distant metastatic recurrence. The 10-year CSS rate was 99.1%. Among the four patients who experienced local recurrence only, all four patients had no SED after additional local treatment. In these four patients, dissection of local recurrence alone was performed in two patients, while completion TT followed by RAI was performed in two patients. Therefore, a total of 11 patients died from FTC or recurrent/persistent SED at the final follow-up.

**Figure 2:**
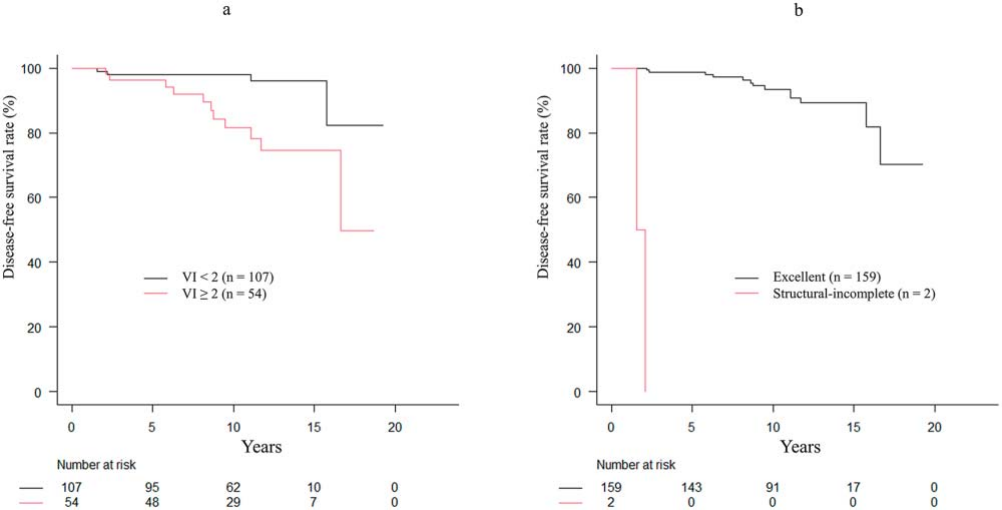
Disease-free survival. (A) The 10-year disease-free survival (DFS) rates of the patients with VI < 2 (*n* = 107) and VI ≥ 2 (*n* = 54) were 98.1 and 81.7%, respectively (*P* = 0.002). (B) The 10-year DFS rates of patients with excellent response (*n* = 159) and structural-incomplete response (*n* = 1) were 96.2% and 0%, respectively (*P* < 0.001).

The 10-year DFS rates of the patients with MI-FTC (*n* = 55), EA-FTC (*n* = 101), and WI-FTC (*n* = 5) were 96.3, 90.3, and 100%, respectively (*P* = 0.735). In addition, the 10-year DFS rates of patients with ATA low- (*n* = 28), intermediate- (*n* = 103), and high-risk (*n* = 30) were 96.4, 91.4, and 92.5%, respectively (*P* = 0.679). Furthermore, the 10-year DFS rates of the patients with VI < 2 (*n* = 107) and VI ≥ 2 (*n* = 54) were 98.1 and 81.7%, respectively (*P* = 0.002) (Fig. 2A).

**Figure 3:**
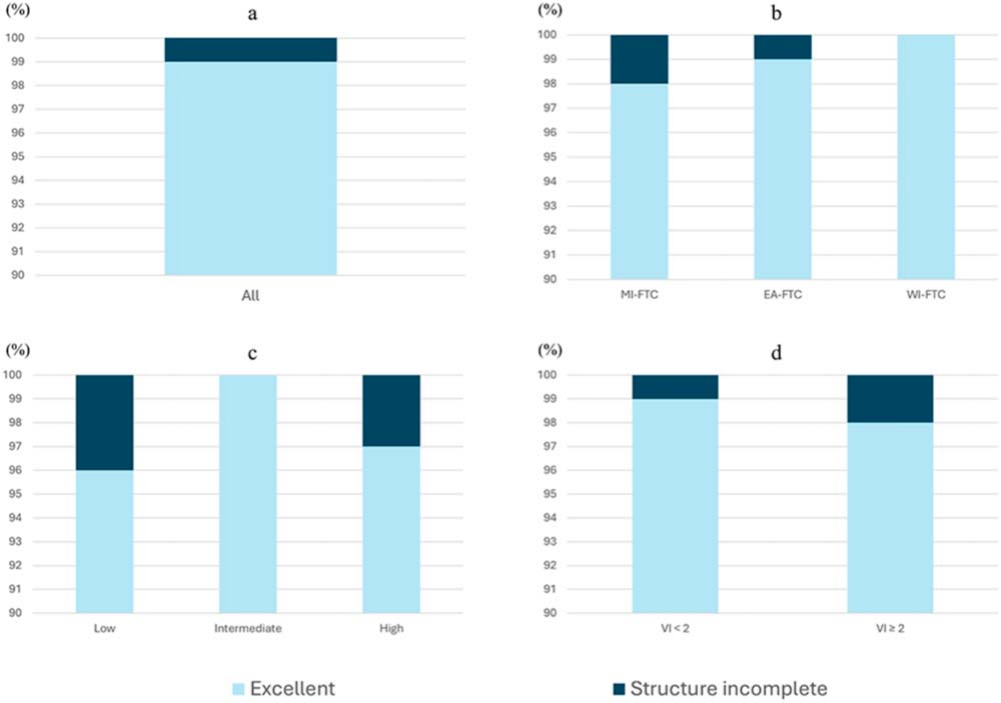
Dynamic risk stratification system. (A) The dynamic risk stratification (DRS) system classified 159 patients (99%) as having an excellent response and 2 (1%) as having a structural-incomplete response. (B) Of the 55 patients with minimally invasive follicular thyroid carcinoma (FTC), 54 (98%) were classified as having an excellent response, and one (2%) had a structural-incomplete response. Of the 101 patients with encapsulated angio-invasive FTC, 100 (99%) were classified as having an excellent response, and one (1%) had a structural-incomplete response. Of the five patients with widely invasive FTC, all five (100%) were classified as having an excellent response. (C) Of the 28 patients with low-risk American Thyroid Association (ATA), 27 (96%) were classified as having an excellent response, and one (4%) had a structural-incomplete response. Of the 103 patients with intermediate-risk ATA, all 103 (100%) were classified as having an excellent response. Of the 30 patients with high-risk ATA, 29 (97%) were classified as having an excellent response, and one (3%) had a structural-incomplete response. (D) Of the 107 patients with vascular invasion (VI) < 2, 106 (99%) were classified as having an excellent response, and one (1%) had a structural-incomplete response. Of the 54 patients with VI ≥ 2, 53 (98%) were classified as having an excellent response, and one (2%) had a structural-incomplete response.

The DRS system classified 159 patients (99%) as having an excellent response and 2 (1%) as having a structural-incomplete response (Fig. 3A and Table 3). The 10-year DFS rates of patients with excellent response and structural-incomplete response were 93.5% and 0%, respectively (*P* < 0.001) (Fig. 2B). Of the 159 patients with excellent response, distant structural disease had been excluded by pre- or post- operative CT.

**Table 3:** Dynamic risk stratification based on response to the initial thyroid surgery.

Response to the initial therapy	*n* (%)	Death from FTC or recurrent/persistent SED, *n* (%)
Excellent	159 (99%)	10/159 (6%)
Structure incomplete	2 (1%)	1/2 (50%)

FTC, follicular thyroid carcinoma; SED, structural evidence of disease.

### Application of DRS in subgroups

- WHO classification.

Application of DRS in WHO classification is shown in Fig. 3B and Supplemental Table 1 (see section on Supplementary materials given at the end of the article). Of the 101 patients with EA-FTC, the 10-year DFS rates of patients with excellent response (*n* = 100), and structural-incomplete response (*n* = 1) were 91.2% and 0%, respectively (*P* < 0.001). As the number of events or patients was small, the comparison of DFS by each response was not performed in patients with MI-FTC and WI-FTC.

(ii) ATA risk stratification

Application of DRS in ATA risk stratification is shown in Fig. 3C and Supplemental Table 2. Of the 103 patients with intermediate-risk ATA, all 103 patients (100%) had excellent response. As the number of patients was small, a comparison of DFS by each response was not performed in patients with low- or high-risk ATA.

(iii) Degree of VI.

**Figure 4:**
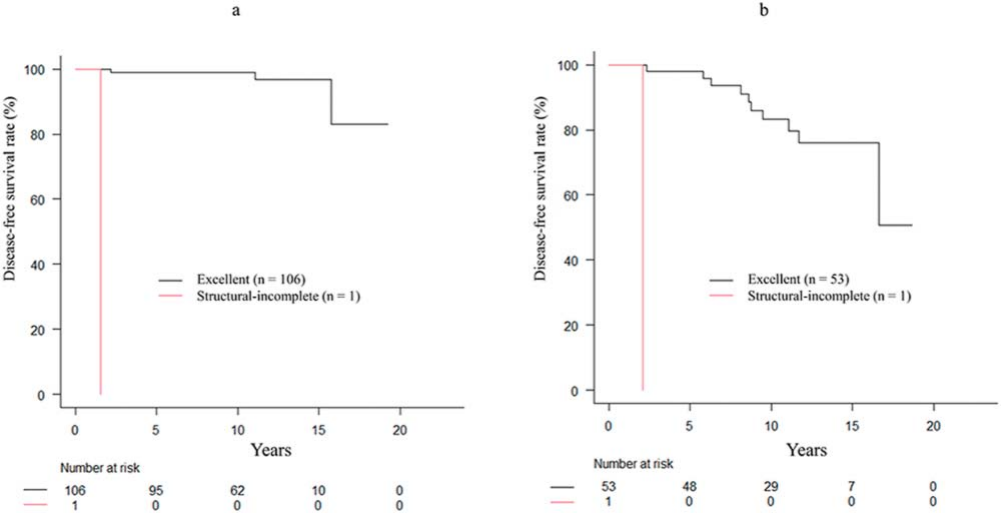
Application of dynamic risk stratification in the degree of vascular invasion. (A) Of the 107 patients with vascular invasion (VI) < 2, the 10-year disease-free survival (DFS) rates of patients with excellent response (*n* = 106), and structural-incomplete response (*n* = 1) were 99.0% and 0%, respectively (*P* < 0.001). (B) Of the 54 patients with VI ≥ 2, the 10-year DFS rates of patients with excellent response (*n* = 53), and structural-incomplete response (*n* = 1) were 83.2% and 0%, respectively (*P* < 0.001).

Application of DRS in degree of VI is shown in Fig. 3D and Table 4. Of the 107 patients with VI < 2, the 10-year DFS rates of patients with excellent response (*n* = 106) and structural-incomplete response (*n* = 1) were 99.0% and 0%, respectively (*P* < 0.001) (Fig. 4A). Of the 106 patients with excellent response, 3 patients experienced disease recurrence. In these three patients, the duration between initial thyroid surgery and recurrence was 2.2, 11.1, and 15.8 years, respectively.

**Table 4:** Degree of vascular invasion and dynamic risk stratification.

Response to the initial therapy	VI < 2 (*n* = 107)		VI ≥ 2 (*n* = 54)	
	*n* (%)	Death from FTC or recurrent/persistent SED, *n* (%)	*n* (%)	Death from FTC or recurrent/persistent SED, *n* (%)
Excellent	106 (99%)	3/106 (3%)	53 (98%)	7/53 (13%)
Structure incomplete	1 (1%)	0	1 (2%)	1/1 (100%)

FTC, follicular thyroid carcinoma; SED, structural evidence of disease; VI, vascular invasion.

Of the 54 patients with VI ≥ 2, the 10-year DFS rates of patients with excellent response (*n* = 53), and structural-incomplete response (*n* = 1) were 83.2% and 0%, respectively (*P* < 0.001) (Fig. 4B). Of the 53 patients with excellent response, 10 patients experienced disease recurrence. In these 10 patients, the duration between initial thyroid surgery and recurrence was 2–5 years in one, 5–8 years in two, 8–10 years in four, and 10 years or more in three patients, respectively.

## Discussion

In this study, we investigated the usefulness of DRS in 161 patients underwent lobectomy alone. Of the 161 patients with FTC, the DRS system classified 159 patients (99%) as having an excellent response and 2 (1%) as having a structural-incomplete response. Furthermore, some FTC patients experienced late recurrence even though they had an excellent response, especially in patients with a higher risk of recurrence (VI ≥ 2).

In the first study on DRS for lobectomy and TT without RAI by Momesso *et al.*, 187 patients who underwent lobectomy and 320 patients with TT without RAI were included (7). In this study, the number of patients who underwent lobectomy was higher than that of TT without RAI, who were excluded. The reason for this may be the differences in histology included in the studies and the treatment approach for each histology. Most patients with FTC initially undergo lobectomy with a diagnosis of follicular or adenomatous nodules. In the study by Momesso *et al.*, most patients had PTC and few had FTC. However, we only included patients with FTC in this study.

The first study of DRS using response to therapy in Asian patients with DTC was validated by Park *et al.* (8). Although the number of patients who underwent lobectomy was relatively large (293 patients), the number of patients with FTC was only 16 (8). To our knowledge, our study had the largest number of FTC patients and may be the first study in which the application of the DRS system was investigated for predicting the prognosis of FTC patients who underwent lobectomy. Given that detailed clinical courses of cause-specific death and recurrence have been reported, the results of this study are useful for determining the appropriate follow-up for individual patients.

In this study, DFS rates were not statistically different in FTC patients stratified according to WHO classification and ATA risk stratification. Patients with MI-FTC with ≤ T2 were defined as ATA low-risk, EA-FTC with VI > 4 and WI-FTC as high-risk, and the others as intermediate-risk. However, it is unclear whether the definition of ATA risk stratification is appropriate. One of the reasons is that WI-FTC histology without VI probably has a good prognosis (13, 17). Therefore, considering all WI-FTCs as high-risk may not be appropriate. In addition, EA-FTC patients with VI = 2 or 3 had relatively more recurrence events because the optimal cutoff value for DFS was a number of VI of 2 in our population (the number of events was six, and all of six FTC patients had VI ≥ 2). Therefore, there is room for improvement in the current ATA risk stratification to evaluate the recurrence risk of FTC.

Our study showed that some FTC patients experienced recurrence after initial thyroid surgery at 5 years or more, even though they had an excellent response. Regarding completion thyroidectomy and RAI therapy for FTC, we usually consider static prognostic factors such as age, histology, degree of VI, and distant metastasis (10). However, some patients do not undergo additional treatment even though they have some risk factors for recurrence (19). Considering that some FTC patients experienced recurrence even though they had an excellent response, long-term follow-up may be needed regardless of response to lobectomy, especially in patients with a higher risk of recurrence.

The present study was associated with several limitations, which were described in detail in our previous study (13). First, the study design was a retrospective cohort study. Second, additional treatment was not performed uniformly; therefore, selection bias might have occurred. Third, the number of slides examined in our study depended on the tumor size and varied among cases. Therefore, some patients with MI-FTC or EA-FTC may have been missed. In addition to the above limitations, we excluded patients who did not have sufficient follow-up data. Furthermore, this study included only 161 FTC patients, and the number of patients may have been small to investigate the utility of the DRS system among FTC patients treated with lobectomy alone. However, lobectomy alone has become much more popular, and it seems important to report on the detailed clinical course of FTC patients after lobectomy (20). This study indicated that long-term follow-up is needed even though FTC patients treated with lobectomy alone achieved an excellent response. Considering the previous report that approximately 90% of recurrence events occurred within 20 years after initial thyroid surgery, follow-up for at least 20 years is probably appropriate (21). Despite these limitations, this study was the largest on DRS among FTC treated with lobectomy alone, and we believe that the results of this study are important for providing appropriate follow-up to individual FTC patients. In addition, our study has some strengths in that we reviewed all cases of FTC according to the latest WHO classification and included detailed information on clinicopathological factors, including the number of VI.

In conclusion, long-term follow-up may be needed in FTC patients treated with lobectomy alone, even though they had an excellent response to initial thyroid surgery, especially in patients with a higher risk of recurrence.

## Supplementary materials



## Declaration of interest

The authors declare that there is no conflict of interest that could be perceived as prejudicing the impartiality of the work reported.

## Funding

This work did not receive any specific grant from any funding agency in the public, commercial, or not-for-profit sector.

## References

[1] Cao W, Qin K, Li F, et al. Comparative study of cancer profiles between 2020 and 2022 using global cancer statistics (GLOBOCAN). J Natl Cancer Cent 2024 4 128–134. (10.1016/j.jncc.2024.05.001) 39282581 PMC11390618

[2] Miranda-Filho A, Lortet-Tieulent J, Bray F, et al. Thyroid cancer incidence trends by histology in 25 countries: a population-based study. Lancet Diabetes Endocrinol 2021 9 225–234. (10.1016/s2213-8587(21)00027-9) 33662333

[3] Ringel MD, Sosa JA, Baloch Z, et al. 2025 American Thyroid Association Management Guidelines for adult patients with differentiated thyroid cancer. Thyroid 2025 35 841–985. (10.1177/10507256251363120) 40844370 PMC13090833

[4] Ito Y, Onoda N & Okamoto T. The revised clinical practice guidelines on the management of thyroid tumors by the Japan Associations of Endocrine Surgeons: core questions and recommendations for treatments of thyroid cancer. Endocr J 2020 67 669–717. (10.1507/endocrj.ej20-0025) 32269182

[5] Pitoia F & Jerkovich F. Dynamic risk assessment in patients with differentiated thyroid cancer. Endocr Relat Cancer 2019 26 R553–R566. (10.1530/erc-19-0213) 31394499

[6] Attia A, Touma E, Lussey-Lepoutre C, et al. Consideration of early dynamic risk stratification to guide discharge from oncologic follow-up in patients with differentiated thyroid cancer. Thyroid 2024 34 1465–1475. (10.1089/thy.2024.0119) 39287064

[7] Momesso DP, Vaisman F, Yang SP, et al. Dynamic risk stratification in patients with differentiated thyroid cancer treated without radioactive iodine. J Clin Endocrinol Metab 2016 101 2692–2700. (10.1210/jc.2015-4290) 27023446 PMC6287503

[8] Park S, Kim WG, Song E, et al. Dynamic risk stratification for predicting recurrence in patients with differentiated thyroid cancer treated without radioactive iodine remnant ablation therapy. Thyroid 2017 27 524–530. (10.1089/thy.2016.0477) 27869547

[9] Baloch ZW, Asa SL, Barletta JA, et al. Overview of the 2022 WHO classification of thyroid neoplasms. Endocr Pathol 2022 33 27–63. (10.1007/s12022-022-09707-3) 35288841

[10] Yamazaki H, Sugino K, Katoh R, et al. Management of follicular thyroid carcinoma. Eur Thyroid J 2024 13 e240146. (10.1530/etj-24-0146) 39419099 PMC11558955

[11] Cho JW, Lee YM, Lee YH, et al. Dynamic risk stratification system in post-lobectomy low-risk and intermediate-risk papillary thyroid carcinoma patients. Clin Endocrinol 2018 89 100–109. (10.1111/cen.13721) 29672893

[12] Lee YM, Cho JW, Hong SJ, et al. Dynamic risk stratification in papillary thyroid carcinoma measuring 1 to 4 Cm. J Surg Oncol 2018 118 636–643. (10.1002/jso.25182) 30114339

[13] Yamazaki H, Sugino K, Katoh R, et al. Role of the degree of vascular invasion in predicting prognosis of follicular thyroid carcinoma. J Clin Endocrinol Metab 2024 109 1291–1300. (10.1210/clinem/dgad689) 38006314

[14] DeLellis RA, Lloyd RV, Heitz PU, et al. Tumours of endocrine organs. In World Health Organization Classification of Tumours: Pathology and Genetics. Lyon: IARC, 2004.

[15] Sugino K, Kameyama K, Ito K, et al. Outcomes and prognostic factors of 251 patients with minimally invasive follicular thyroid carcinoma. Thyroid 2012 22 798–804. (10.1089/thy.2012.0051) 22853727

[16] Yamazaki H, Katoh R, Sugino K, et al. Encapsulated angioinvasive follicular thyroid carcinoma: prognostic impact of the extent of vascular invasion. Ann Surg Oncol 2022 29 4236–4244. (10.1245/s10434-022-11401-x) 36138288

[17] Yamazaki H, Sugino K, Katoh R, et al. New insights on the importance of the extent of vascular invasion in widely invasive follicular thyroid carcinoma. World J Surg 2023 47 2767–2775. (10.1007/s00268-023-07127-w) 37516689

[18] Kanda Y. Investigation of the freely available easy-to-use software ‘EZR’ for medical statistics. Bone Marrow Transplant 2013 48 452–458. (10.1038/bmt.2012.244) 23208313 PMC3590441

[19] Yamazaki H, Sugino K, Katoh R, et al. Outcomes for minimally invasive follicular thyroid carcinoma in relation to the change in age stratification in the AJCC 8th edition. Ann Surg Oncol 2021 28 3576–3583. (10.1245/s10434-020-09397-3) 33237449

[20] Robenshtok E, Bachar G & Ritter A. Approach to the patient with thyroid cancer: selection and management of candidates for lobectomy. J Clin Endocrinol Metab 2025 110 e2327–e2337. (10.1210/clinem/dgae903) 39745824

[21] Dong W, Horiuchi K, Tokumitsu H, et al. Time-varying pattern of mortality and recurrence from papillary thyroid cancer: lessons from a long-term follow-up. Thyroid 2019 29 802–808. (10.1089/thy.2018.0128) 30931815

