# Supplementary material for: Dynamic risk stratification in patients with follicular thyroid carcinoma treated with lobectomy
Source: Eur Thyroid J. 2025 Nov 17;14(6):e250154. doi: 10.1530/ETJ-25-0154 (PMC12623134; doi:10.1530/ETJ-25-0154)
Supplement: Supplementary file 1 [file supplementary_materials.pdf]

Supplemental Table 1. WHO classification and dynamic risk stratification

	MI-FTC (n = 55)		EA-FTC (n = 101)		WI-FTC (n = 5)	
	n (%)	Death from FTC or Recurrent/Persistent SED, n (%)	n (%)	Death from FTC or Recurrent/Persistent SED, n (%)	n (%)	Death from FTC or Recurrent/Persistent SED, n (%)
Response to the initial therapy						
Excellent	54 (98%)	3 (6%, 3/54)	100 (99%)	7 (7%, 7/100)	5 (100%)	0
Structure incomplete	1 (2%)	0	1 (1%)	1 (100%, 1/1)	0	0

EA, encapsulated angio-invasive; FTC, follicular thyroid carcinoma; MI, minimally invasive; SED, structural evidence of disease; WHO, World Health Organization; WI, widely invasive

Supplemental Table 2. ATA 2025 risk stratification and dynamic risk stratification

	Low-risk (n = 28)		Intermediate-risk (n = 103)		High-risk (n = 30)	
	n (%)	Death from FTC or Recurrent/Persistent SED, n (%)	n (%)	Death from FTC or Recurrent/Persistent SED, n (%)	n (%)	Death from FTC or Recurrent/Persistent SED, n (%)
Response to the initial therapy						
Excellent	27 (96%)	1 (4%, 1/27)	103 (100%)	5 (5%, 5/103)	29 (97%)	4 (14%, 4/29)
Structure incomplete	1 (4%)	0	0	0	1 (3%)	1 (100%, 1/1)

ATA, American Thyroid Association; FTC, follicular thyroid carcinoma; SED, structural evidence of disease
